# Highly contiguous draft nuclear genome assembly of *Teratoramularia* sp. strain RD051456 isolated from plant debris in Yamanashi Prefecture, Japan

**DOI:** 10.1128/mra.00921-26

**Published:** 2026-09-24

**Authors:** Masataka Izumi, Tomomasa Oka

**Affiliations:** 1 Graduate School of Agriculture, Kyoto University 12918, Kyoto-shi, Kyoto, Japan; 2 National Institute of Global Health and Medicine, Japan Institute for Health Security 739298 , Shinjuku-ku, Tokyo, Japan; USDA-ARS San Joaquin Valley Agricultural Sciences Center, Parlier, California, USA

**Keywords:** *Teratoramularia*, PacBio HiFi sequencing, fungal genomics, draft genome assembly, Dothideomycetes

## Abstract

We report a highly contiguous draft nuclear genome assembly of *Teratoramularia* sp. strain RD051456, isolated from plant debris in Yamanashi Prefecture, Japan. PacBio HiFi sequencing and hifiasm assembly produced a 26.26-Mb nuclear genome comprising 17 contigs, providing a resource for taxonomic, phylogenetic, and comparative genomic studies of *Teratoramularia*.

## ANNOUNCEMENT

*Teratoramularia* is a genus of plant-associated ramularia-like fungi belonging to the class Dothideomycetes and family Teratosphaeriaceae, including *Teratoramularia rumicicola* strains with potential for microbial weed control (1–3). Genomic resources for this genus remain limited (4). Strain RD051456, originally isolated from plant debris in Yamanashi Prefecture, Japan, was obtained from the Biological Resource Center, National Institute of Technology and Evaluation (NBRC, NITE), Japan (5). Detailed sampling information and geographic coordinates were unavailable from the repository. In the NITE Biological Resource Data Platform, the strain was assigned to the genus *Teratoramularia* based on a 537-bp LSU rDNA D1/D2 sequence under data ID ANGE0000500001353 (6). We report a highly contiguous draft nuclear genome assembly of strain RD051456 generated from PacBio HiFi reads.

Strain RD051456 was cultured on potato dextrose agar (AccuDia; Shimadzu Diagnostics Corporation) at 25°C in the dark for 21 days. Mycelia were stored at −80°C until DNA extraction. High-molecular-weight genomic DNA was extracted from mycelia ground in liquid nitrogen and purified using a Genomic-tip 20/G column (Qiagen), followed by purification using Short Read Eliminator XS (PacBio). No mechanical shearing was performed prior to library preparation. A polymerase chain reaction (PCR)-amplified SMRTbell library was prepared using the SMRTbell Prep Kit 3.0 (PacBio) and Twist Universal Adapter System (Twist Bioscience), size-selected using diluted AMPure PB beads to deplete fragments < 5 kb, and sequenced on a PacBio Revio system using the Revio SPRQ polymerase kit. HiFi reads were generated using SMRT Link v25.2.0.266456 (7), with CCS reads having mean quality values below Q20 discarded. PCR adapter sequences and PCR duplicate reads were removed using lima v2.12.0 (8) and pbmarkdup v1.0.3 (9), respectively. A total of 131,607 HiFi reads comprising 946,647,051 bp were obtained, with a read N50 of 7,785 bp and an estimated coverage of 36.0×. The reads were assembled using hifiasm v0.25.0-r726 (10) with the -l 0 option; otherwise, default parameters were used. Four low-GC contigs representing overlapping putative mitochondrial assemblies were excluded based on assembly graph inspection and BLASTN searches against the NCBI core nucleotide database (11). The terminal 100 bp of each nuclear contig were examined for tandem repeats of the canonical telomeric motifs CCCTAA and TTAGGG. A contig end was considered telomeric when at least three consecutive exact copies of either motif were present within the terminal 100 bp.

Assembly statistics for the nuclear genome assembly were calculated using QUAST v5.3.0 (12). The assembly comprised 17 contigs with a total length of 26,260,140 bp, an N50 value of 4,317,038 bp, and a maximum contig length of 4,910,207 bp. The GC content was 51.82%, and the assembly contained no ambiguous bases. Canonical telomeric repeats were observed at both ends of eight contigs and at one end of five additional contigs. Genome completeness was assessed using BUSCO v6.1.0 (13) in the genome mode with MetaEuk (14) and the dothideomycetes_odb12.2 data set. Of the 4,064 BUSCO groups examined, 94.1% were complete, including 93.8% single-copy and 0.2% duplicated BUSCOs; 0.8% were fragmented, and 5.1% were missing. The genome assembly was not annotated.

## Data Availability

This Whole Genome Shotgun project has been deposited at DDBJ/ENA/GenBank under the accession number JCBNUD000000000 (BioProject PRJNA1497651; BioSample SAMN61833673). The version described in this paper is version JCBNUD010000000. Raw PacBio HiFi sequencing reads are available in the NCBI Sequence Read Archive under accession number SRR39795162.
